# The Effects of Downloading a Government-Issued COVID-19 Contact Tracing App on Psychological Distress During the Pandemic Among Employed Adults: Prospective Study

**DOI:** 10.2196/23699

**Published:** 2021-01-12

**Authors:** Norito Kawakami, Natsu Sasaki, Reiko Kuroda, Kanami Tsuno, Kotaro Imamura

**Affiliations:** 1 Department of Mental Health Graduate School of Medicine The University of Tokyo Tokyo Japan; 2 Division for Environment, Health and Safety The University of Tokyo Tokyo Japan; 3 School of Health Innovation Kanagawa University of Human Services Kawasaki Japan

**Keywords:** coronavirus disease, digital contact tracing, mental health, working population, longitudinal study, COVID-19, contact tracing, surveillance, tracking, anxiety, distress

## Abstract

**Background:**

Downloading a COVID-19 contact tracing app may be effective in reducing users’ worry about COVID-19 and psychological distress.

**Objective:**

This 2.5-month prospective study aimed to investigate the association of downloading a COVID-19 contact tracing app, the COVID-19 Contact Confirming Application (COCOA), released by the Japanese government, with worry about COVID-19 and psychological distress in a sample of employed adults in Japan.

**Methods:**

A total of 996 full-time employed respondents to an online survey conducted May 22-26, 2020 (baseline), were invited to participate in a follow-up survey August 7-12, 2020 (follow-up). A high level of worrying about COVID-19 and high psychological distress were defined by baseline and follow-up scores on a single-item scale and the Kessler 6 (K6) scale, respectively. The app was released between the two surveys, on June 17. Participants were asked at follow-up if they downloaded the app.

**Results:**

A total of 902 (90.6%) of 996 baseline participants responded to the follow-up survey. Among them, 184 (20.4%) reported that they downloaded the app. Downloading of the contact tracing app was significantly negatively associated with psychological distress at follow-up after controlling for baseline variables, but not with worry about COVID-19.

**Conclusions:**

This study provides the first evidence that using a government-issued COVID-19 contact tracing app may be beneficial for the mental health of employed adults during the COVID-19 pandemic.

## Introduction

Contact tracing is one of the most effective methods of controlling infectious diseases. During the 2020 COVID-19 pandemic, many smartphone apps for digital contact tracing have been developed and widely used [1-3]. These contact tracing smartphone apps automatically record contacts with other people through smartphones during everyday life. A user will be notified if someone he/she contacted recently had a positive test result for COVID-19 infection, and be encouraged to take a test for COVID-19. The use of these apps is considered acceptable by a majority of the public [4], despite ethical concerns about people’s privacy [5]. Such apps are expected to slow the transmission of COVID-19 [6], although more research is required to confirm their effectiveness. Psychological distress was reported to increase in the community during the nationwide spread of COVID-19 [7-10], partly due to fear of COVID-19 infection [7,9]. Poor mental health has been considered another public health problem related to the COVID-19 outbreak [9]. As a contact tracing app could help users identify their own risk of infection with COVID-19, users can be assured that they are at low risk when no notification comes from the system, indicating that they have not had close contact with people infected by COVID-19. Thus, the use of a COVID-19 contact tracing app may be effective at reducing fear of and worry about COVID-19 and reducing users’ psychological distress. However, to date, no study has reported on the mental health effects of using a COVID-19 contact tracing app.

This prospective study aimed to examine the effect of the use of a COVID-19 contact tracing app released by the Japanese government in mid-June 2020 on the perceived threat from COVID-19 and psychological distress in a sample of employed adults in Japan. We examined the association between reported use of the COVID-19 contact tracing app and worry about COVID-19 and psychological distress at follow-up, adjusting for these variables at baseline.

## Methods

### Study Design and Participants

This study was a 2.5-month prospective study with two consecutive online surveys. Participants responded to an online “closed” survey conducted May 22-26, 2020 (baseline), which was part of a larger longitudinal study of full-time employees aged 20-59 years that were recruited by an internet survey company (Macrimill Inc) from a large pool (>1,300,000) of self-selected preregistered community-dwelling residents across Japan [11,12]. After excluding 36 respondents who were unemployed at baseline, as their financial or health condition may confound study findings, a total of 996 respondents were invited via email to participate in a follow-up survey on August 7-12, 2020 (follow-up). Between the two surveys, Japan experienced the second wave of the outbreak; in 78 days (May 27-August 12), the number of new COVID-19 cases per day increased from 22 (on June 8) to a maximum of 1595 (August 7), with an average of 440 per day [13]. All surveys were made using online questionnaires on a website that was specifically designed for the surveys and required an ID and password to log in. The survey system allowed each respondent to submit a questionnaire only once. Each questionnaire consisted of 73 questions displayed on one page, with automated completeness checks before the questionnaire was submitted; no review step was provided. The participants were given a small incentive (a token equivalent to 30 JPY [US $0.29]) for completing each questionnaire. Study participants were informed about the purpose of the study, the investigators, the length of the questionnaires, and processes related to the data (anonymization, place and duration of storage), and participated in the surveys voluntarily.

### Use of the COVID-19 Contact Tracing App

On June 19, 2020, the Japanese government released a free contact tracing app for COVID-19, called the COVID-19 Contact Confirming Application (COCOA), for iOS and Android [14]. The app does not collect personal information such as phone numbers or location. Rather, the app records encounters with other phones that were within one meter and lasted for more than 15 minutes as encrypted data. The users are notified when a user who they have come into close contact with for more than 15 minutes reports a positive test result for COVID-19; the notification only occurs after the contact enters the positive result in the app and allows the result to be shared to other users anonymously. In addition, the validity of the app measuring the information was not reported and thus is unclear. The app had been downloaded 13.2 million times by August 14, 2020 [14]. By then, 252 (0.002%) users were confirmed to have COVID-19. Participants were asked if they downloaded this app (yes or no) at follow-up [14].

### Measures

#### Worry About COVID-19

We used a single-item scale to measure worry about COVID-19 at baseline and follow-up, by asking “Do you worry about COVID-19?” and using a 6-point Likert-type response scale [11,12]. The responses were dichotomized into high (“strongly” to “somewhat positive”) and low (“somewhat negative” to “not at all”).

#### Psychological Distress

Psychological distress (depression and anxiety) in the last 30 days was measured using the Kessler 6 (K6) scale [15] at baseline and follow-up. Acceptable levels of reliability and validity of the Japanese version have been reported [16,17]. Psychological distress was defined as having a K6 score of ≥5, corresponding to a mild level of distress, according to a previous study [17]. For a sensitivity analysis, we also used severe psychological distress (K6 score ≥13) as an alternative outcome [18].

#### Demographic Variables

Participants were asked their sex (male or female), age (<35 years, 35-49 years, or ≥50 years), marital status (currently married or not), educational attainment (high school graduate and lower or university graduate and higher), and occupation (managers, non–manual workers, manual workers in non–health care settings, or health care workers); whether they work from home (yes including partially or never), live with a child up to high school age (yes or no), or live in high-risk areas designated during the COVID-19 emergency between April and May 2020 by the government (yes or no) [19]; and if they had any chronic physical condition (any of 10 predetermined conditions) at baseline.

### Statistical Analysis

The adjusted prevalence of worry about COVID-19 and high psychological distress at follow-up (controlling for baseline levels) were compared between those who did or did not use the COVID-19 contact tracing app (Mantel-Haenszel chi-square test). Multiple logistic regression analyses were conducted to estimate the effect (odds ratio [OR] and 95% CI) of worry about COVID-19 or high psychological distress at follow-up, adjusting for the baseline demographic variables and baseline values of these variables. No weighting was made to adjust for the nonrepresentativeness of the sample. Statistical significance was set at *P*<.05. SPSS (Version 26.0; IBM Corp) was used for analyses.

### Ethical Considerations

Online informed consent was obtained from all participants with full disclosure and explanation of the purpose and procedures of this study. We explained that their participation was voluntary, and that they could withdraw consent for any reason, simply by not completing the questionnaire. This study was approved by the Research Ethics Committee of the Graduate School of Medicine/Faculty of Medicine at The University of Tokyo [number 10856-(2)(3)(4)(5)].

## Results

A total of 902 (90.6%) of 996 participants at baseline responded to the follow-up survey. Among them, 184 (20.4%) reported that they downloaded the app. The respondents who downloaded the app were significantly more likely to be male, older, living with a child, university graduates or higher, and working from home than respondents who did not download the app (Table 1). Prevalence of high psychological distress was slightly but not significantly greater among app users at baseline, but the pattern was reversed at follow-up. Compared to the national labor force statistics in Japan, the sample was less represented by manual workers, while sex and age distributions were similar.

Downloading of the app was significantly negatively associated with psychological distress at follow-up after adjusting for psychological distress at baseline (adjusted prevalence of 42.2% and 49.6% for users and nonusers, respectively; OR 0.61, 95% CI 0.39-0.93; *P*=.02). Downloading of the contact tracing app was significantly negatively associated with psychological distress at follow-up in the group without high psychological distress at baseline (*P*=.04; Multimedia Appendix 1). Downloading of the app was not significantly associated with worry about COVID-19 at follow-up after adjusting for worry about COVID-19 at baseline (adjusted prevalence of 61.9% and 61.9%, respectively; OR 1.01, 95% CI 0.68-1.51; *P*=.96). Downloading of the app was significantly negatively associated with psychological distress at follow-up after adjusting for all covariates (OR 0.59, 95% CI 0.38-0.91; *P*=.02; Table 2). Downloading of the app was not significantly associated with worry about COVID-19 (OR 1.25, 95% CI 0.65-2.40; *P*=.50).

We conducted these multivariate analyses using age with a different categorization (20-29 years old, 30-39 years old, 40-49 years old, and 50-59 years old) and as a continuous variable, and obtained similar findings. When using the alternative definition of severe psychological distress (ie, K6 score of ≥13), downloading of the app was not significantly associated with severe psychological distress at follow-up (adjusted prevalence of 13.0% and 13.2% for users and nonusers, respectively; OR 0.88, 95% CI 0.54-1.44; *P*=.71) or after adjusting for all covariates (OR 0.95, 95% CI 0.54-1.69; *P*=.92).

**Table 1:** Demographic characteristics at baseline and psychological variables at baseline and follow-up among respondents who used or did not use the COVID-19 COntact COnfirming Application (COCOA) at follow-up.

Variables		Total sample (N=902), n (%)		Nonusers (n=718), n (%)		App users (n=184), n (%)		*P* value^a^		National Labour Force Survey^b^, %	
Sex (female)		434 (48.1)		361 (50.2)		73 (39.7)		.001		45.2	
**Age, years**											
	20-34		262 (29.0)		224 (31.2)		38 (20.7)		.01		30.3
	35-49		375 (41.6)		294 (40.9)		81 (44.0)		—^c^		43.4
	50-60		265 (29.4)		200 (27.9)		65 (35.3)		—		26.3
Marital status (married)		662 (73.4)		525 (73.1)		137 (74.5)		.78		ND^d^	
Living with a child (yes)		228 (25.3)		167 (23.3)		61 (33.2)		.008		ND	
Education (university or higher)		476 (52.8)		358 (49.9)		118 (64.1)		.001		ND	
**Occupation**											
	Managers		93 (10.3)		66 (9.2)		27 (14.7)		.16		1.3
	Non–manual workers		480 (53.2)		384 (53.5)		96 (52.2)		—		35.5
	Manual workers		232 (25.7)		190 (26.5)		42 (22.8)		—		57.7
	Health care workers		97 (10.8)		78 (10.9)		19 (10.3)		—		5.6
Working from home (yes)		298 (33.0)		213 (29.7)		85 (46.2)		<.001		ND	
Chronic condition (any)		298 (33.0)		213 (29.7)		85 (46.2)		.29		ND	
Living in prior high-risk areas (yes)		635 (70.4)		508 (70.8)		127 (69.0)		.65		ND	
**Worry about COVID-19**											
	At baseline		513 (56.9)		403 (56.1)		110 (59.8)		.40		ND
	At follow-up		559 (62.0)		442 (61.60		117 (63.6)		.67		ND
**Psychological distress (K6 score of ≥5)**											
	At baseline		414 (45.9)		323 (45.0)		91 (49.5)		.28		ND
	At follow-up		434 (48.1)		352 (49.0)		82 (44.6)		.28		ND

^a^Based on the chi-square test.

^b^Proportions among employed adults aged 20-59 years (N=51.8 million) based on the Japan National Labour Force Survey 2019.

^c^—: not available.

^d^ND: no data.

**Table 2:** The association between use of the COVID-19 contact tracing app and worry about COVID-19 and high psychological distress at follow-up, adjusting for covariates at baseline.

Variables		Worry about COVID-19 (high)		High psychological distress (K6 score of ≥5)	
		Odds ratio (95% CI)	*P* value	Odds ratio (95% CI)	*P* value
Use of the contact tracing app (yes)		1.25 (0.65-2.40)	.50	0.59 (0.38-0.91)	.02^a^
Sex (female)		2.77 (1.56-4.93)	.001^a^	1.13 (0.77-1.67)	.52
**Age (years)**					
	20-34	1.00	N/A^b^	1.00	N/A
	35-49	1.12 (0.63-1.99)	.71	1.21 (0.79-1.86)	.38
	50-60	2.44 (1.16-5.15)	.02^b^	0.98 (0.60-1.59)	.94
Marital status (married)		0.63 (0.34-1.15)	.13	1.54 (1.02-2.33)	.04^b^
Living with a child (yes)		1.22 (0.65-2.27)	.54	1.16 (0.75-1.80)	.50
Education (university or higher)		1.10 (0.63-1.92)	.75	1.10 (0.75-1.61)	.64
**Occupation**					
	Managers	1.00	N/A	1.00	N/A
	Non–manual workers	1.03 (0.44-2.41)	.95	1.83 (0.95-3.53)	.07
	Manual workers	1.37 (0.51-3.70)	.54	1.96 (0.95-4.03)	.07
	Health care workers	1.02 (0.32-3.24)	.97	2.54 (1.10-5.84)	.03^b^
Working from home (yes)		1.60 (0.90-2.84)	.11	1.30 (0.87-1.96)	.20
Chronic condition (any)		1.12 (0.56-2.23)	.75	1.62 (1.03-2.55)	.04^b^
Living in prior high-risk areas (yes)		0.56 (0.31-1.03)	.06	0.80 (0.54-1.18)	.25
**Baseline psychological status**					
	COVID-19 anxiety	3.03 (0.26-135.83)	<.001^a^	N/A	N/A
	Psychological distress (K6 score of ≥5)	N/A	N/A	20.64 (14.39-29.60)	<.001^a^

^a^*P*<.05.

^b^N/A: not applicable.

## Discussion

### Principal Findings

This study found that downloading of the COVID-19 contact tracing app released by the Japanese government was significantly negatively associated with psychological distress in a sample of employed adults in Japan. This is the first evidence indicating that a COVID-19 contact tracing app may be beneficial for people’s mental health during the COVID-19 pandemic. Unexpectedly, downloading of the COVID-19 contact tracing app did not significantly influence worry about COVID-19. This may be interpreted as the users of the app becoming better able to cope with worry about COVID-19 (a stressor) and reduce their psychological distress, even though they still worry.

A contact tracing app decreased the prevalence of mild psychological distress by 7.4 points on average (a 15% reduction from its prevalence among nonusers). The effect size was rather small, but it might have a large impact on mental health (which has been reported to have deteriorated during the pandemic [7-9]) because there are a large number of potential users. However, we could not replicate the finding when using severe psychological distress (K6 scores of ≥13) as an indicator of poor mental health. This is partly attributable to the small sample size, with a lower prevalence of severe psychological distress than mild physical distress in the sample. Another possibility is that the effect of the app may be less clear for those with a severe level of psychological distress or recovering from it. This corresponds to our observation that downloading the app was significantly negatively associated with high psychological distress at follow-up only in the group without high psychological distress at baseline. The present finding should be replicated with mental health indicators measuring different levels of distress severity in a larger sample. The mechanism underlying the negative association between downloading the app and psychological distress is unclear. Downloading the app itself may serve as an attempt at active coping against the threat of COVID-19 infection for the users, which could improve their sense of control or self-efficacy and reduce psychological distress [20]. Another possibility is that users may have a better personal relationship with family, friends, or colleagues as a result of downloading the app because the users could be seen as “trustworthy” by those around them due to them taking preventive measures (ie, using the app). This may be quite important in circumstances where physical distancing is encouraged and discrimination and stigma related to COVID-19 are increased [20]. Improved social relationships may lead people to have better social support, and thus their psychological distress could be reduced. The psychological effects of downloading a COVID-19 contact tracing app as well as the underlying mechanisms behind those effects should be investigated using relevant psychological theories and scales in future research.

While the app certainly provides users with objective information about the risk of COVID-19 infection, participants’ worry about COVID-19 (ie, perception of the risk of infection) was not different at follow-up between users of the contact tracing app and nonusers. In fact, the number of notifications that users had been in contact with a COVID-19–positive person was very limited (only 0.002% of users registered as having COVID-19 as of August 14, 2020, in the case of the COCOA app) [14]. However, users who do not receive contact notifications may not be assured that they have a low risk of being infected because there are many other opportunities for infection that the app cannot inform them about. This finding is consistent with a previous report indicating that implementing measures to protect against COVID-19 in the workplace was not associated with a reduction in the perceived risk of COVID-19 infection [11]. The effect of using the app may not be strong enough to decrease uncertainty [21] and change the perceived risk of infection.

Although this study indicated a possible benefit to mental health related to using a contact tracing app, there are other issues to be considered before encouraging the use of the app [21]. There are still privacy concerns because the anonymization process may not be perfect; it is unclear how long the collected data will be stored; and the system may be vulnerable to a cyberattack [5,22]. There are also concerns about the transparency and accuracy of such apps; it is hard for people to judge the quality and trustworthiness of the apps, even if the source code is published. Therefore, the apps should be pretested to indicate how accurately they measure proximity to and meaningful contacts with positive cases [22]. It is necessary to investigate the extent to which privacy concerns and possible measurement errors are ethically and psychologically acceptable. There is also concern about unintended side effects. Related to our study finding, for instance, the apps may increase the psychophysiological arousal of users as they continuously await the arrival of a notification, which could lead to physical complaints and sleep problems. Such an adverse effect of downloading this type of app should be investigated and if adverse effects exist, warnings should be communicated to the users. As all these issues could influence the use of the app itself, as well as the possible mental health benefit of using the apps, future research should be conducted to fully explore the ethical and behavioral aspects of the use of a contact tracing app.

### Limitations

First, although the study employed a prospective design, downloading of the contact tracing app was measured at follow-up. Thus, the direction of causality between use of the app and psychological distress is unclear; it is conceivable that participants whose mental health has improved are more likely to start using the app. In addition, users had to complete a few steps after downloading the app to approve the use of the app and allow it to access the Bluetooth function. Thus, downloading the app may not necessarily be equal to use of the app. Some may have uninstalled the app soon after they downloaded it. Research using a more accurate assessment of the use of the app is needed to confirm our findings. Second, we investigated the effect of a government-released contact tracing app in this study. The findings may not be applicable to similar contact tracing apps released by commercial entities, which may be less trusted by people than government-released ones. Third, because the study sample included only employees, and might be demographically biased in some other way, the findings may not be generalizable to the general population (eg, they might not apply to unemployed individuals who might be experiencing financial hardship or have severe health conditions). The prevalence of psychological distress in this sample (40%-50%) was slightly higher than that reported in previous studies (34%-38%) [10]. The sample may be more distressed than the general population. Furthermore, the finding may not be generalizable to other countries with different policies, cultural norms, and behaviors (such as wearing masks) in response to the COVID-19 pandemic. The risk of infection and mortality may also be different from other countries, as Japan had a less stringent policy on social distancing (eg, lockdown), a higher proportion of people wearing masks [23], and a lower proportion of confirmed cases per capita [13] during the study period. Fourth, while the sampling ratio of the respondents to the total employed population of Japan was extremely small, it is unlikely that some respondents were from the same family, social group, or workplace. However, if that was the case, it could result in an overestimation of the effect of the app. Fifth, behavioral patterns underlying the use of the app [24,25] and social desirability may confound the findings. For instance, participants may respond favorably to both questions about downloading the app and psychological distress. A relationship between the motivation to use the app or the duration of app use and psychological distress was not investigated in this study. The living environment, family environment, and social environment of the respondents may confound the findings. We could not investigate differences in the effect of using the app among various demographic groups, such as sex, age, and educational attainment, due to the small sample size. Future research should address these issues.

### Conclusion

This prospective study found that downloading of the COVID-19 contact tracing app released by the Japanese government was significantly negatively associated with psychological distress in a sample of employed adults in Japan. Downloading of a COVID-19 contact tracing app may reduce psychological distress among users during the COVID-19 pandemic. However, other ethical and psychological issues related to contact tracing apps need to be fully discussed and resolved before recommending the app to public health officials.

## Appendix

Multimedia Appendix 1 Supplementary table.

## Abbreviations

K6: Kessler 6
OR: odds ratio
