# Supplementary material for: The Effects of Downloading a Government-Issued COVID-19 Contact Tracing App on Psychological Distress During the Pandemic Among Employed Adults: Prospective Study
Source: JMIR Ment Health. 2021 Jan 12;8(1):e23699. doi: 10.2196/23699 (PMC7806338; doi:10.2196/23699)
Supplement: Multimedia Appendix 1 [file mental_v8i1e23699_app1.docx]

Multimedia Appendix 1. Comparison of adjusted prevalence of worry about COVID-19 and psychological distress controlling for the baseline values among respondents who did or did not download the COVID-19 contact tracing app “COCOA” at follow-up.

| Prevalence of worry about COVID-19 at follow-up: | | | | | | | | |
| --- | --- | --- | --- | --- | --- | --- | --- | --- |
|  | Did not download the app | | | Downloaded the app | | |  |  |
|  | N | No. of case | % | N | No. of case | % |  | *P* |
| Worry about COVID-19 at baseline: | | | |  |  |  |  |  |
| Low | 315 | 107 | 34.0 | 74 | 21 | 28.4 |  | .41 |
| High | 403 | 335 | 83.1 | 110 | 96 | 87.3 |  | .38 |
| Total | 718 | 442 | 61.6 | 184 | 117 | 63.6 |  | .67 |
| Baseline-adjusted prevalence (%) | | | 61.9 |  |  | 61.9 |  | .96† |
| Prevalence of psychological distress at follow-up: | | | | | | | | |
|  | Did not download the app | | | Downloaded the app | | |  |  |
|  | N | No. of case | % | N | No. of case | % |  | *P* |
| Psychological distress at baseline: | | | |  |  |  |  |  |
| Low | 395 | 83 | 21.0 | 93 | 11 | 11.8 |  | .04 |
| High | 323 | 269 | 83.3 | 91 | 71 | 78.0 |  | .17 |
| Total | 718 | 352 | 49.0 | 184 | 82 | 44.6 |  | .28 |
| Baseline-adjusted prevalence (%) | | | 49.6 |  |  | 42.2 |  | .02‡ |

† Mantel- Haenszel OR 1.01; 95% CI 0.68-1.51.

‡ Mantel-Haenszel OR 0.61; 95% CI 0.39-0.93.
